# PAQR6 Upregulation Is Associated with AR Signaling and Unfavorite Prognosis in Prostate Cancers

**DOI:** 10.3390/biom11091383

**Published:** 2021-09-18

**Authors:** Min Yang, Jean Chong Li, Chang Tao, Sa Wu, Bin Liu, Qiang Shu, Benyi Li, Runzhi Zhu

**Affiliations:** 1 The Children’s Hospital, Zhejiang University School of Medicine, National Clinical Research Center for Child Health, Hangzhou 310052, China; yangmin2018@zju.edu.cn (M.Y.); taoc777@163.com (C.T.); 2 Cancer Center, Zhejiang University, Hangzhou 310058, China; 3 Department of Urology, The University of Kansas Medical Center, Kansas City, KS 66160, USA; jeanli2000@yahoo.com (J.C.L.); sawsa@126.com (S.W.); 4 Laboratory of Hepatobiliary Surgery, The Affiliated Hospital of Guangdong Medical University, Zhanjiang 524001, China; binliu831201@163.com

**Keywords:** PAQR6, prostate cancer, patient survival, disease progression, androgen deprivation therapy

## Abstract

Progesterone-induced rapid non-genomic signaling events have been confirmed through several membrane progesterone receptors (mPR). Some mPRs were reported to correlate with cancer progression and patient prognosis. In this study, we conducted a comprehensive analysis of all progesterone receptor (PGR)-related genes in prostate cancer tissues and examined the correlations of their expression levels with disease progression and patient survival outcomes. We utilized multiple RNA-seq and cDNA microarray datasets to analyze gene expression profiles and performed logistics aggression and Kaplan-Meier survival analysis after stratifying patients based on tumor stages and Gleason scores. We also used NCBI GEO datasets to examine gene expression patterns in individual cell types of the prostate gland and to determine the androgen-induced alteration of gene expression. Spearman coefficient analysis was conducted to access the correlation of target gene expression with treatment responses and disease progression status. The classic PGR was mainly expressed in stromal cells and progestin and adipoQ receptor (PAQR) genes were the predominant genes in prostate epithelial cells. Progesterone receptor membrane component-1 (PGRMC1) was significantly higher than PGRMC2 in all prostate cell types. In prostate cancer tissues, PAQR6 expression was significantly upregulated, while all other genes were largely downregulated compared to normal prostate tissues. Although both PAQR6 upregulation and PAQR5 downregulation were significantly correlated with tumor pathological stages, only PAQR6 upregulation was associated with Gleason score, free-prostate-specific antigen (fPSA)/total-PSA (tPSA) ratio, and patient overall survival outcomes. In addition, PAQR6 upregulation and PGR/PGRMC1 downregulation were significantly associated with a quick relapse. Conversely, in neuroendocrinal prostate cancer (NEPC) tissues, PAQR6 expression was significantly lower, but PAQR7/8 expression was higher than castration-resistant prostate cancer (CRPC) tissues. PAQR8 expression was positively correlated with androgen receptor (AR) score and AR-V7 expression levels but inversely correlated with NEPC score in metastatic CRPC tumors. This study provides detailed expression profiles of membrane progesterone receptor genes in primary cancer, CRPC, and NEPC tissues. PAQR6 upregulation in primary cancer tissues is a novel prognostic biomarker for disease progression, overall, and progression-free survival in prostate cancers. PAQR8 expression in CRPC tissues is a biomarker for AR activation.

## 1. Introduction

Progesterone is a female hormone in modulating reproductive activities through the classical nuclear progesterone receptors, and these physiological functions of progesterone are primarily confined to ovulation during the menstrual cycle and to pregnancy [1]. Although there were no significant differences in progesterone serum levels between adult males (1.21 ± 0.41 nM) and postmenopausal females (1.24 ± 1.18 nM), little is known about the physiological functions of progesterone in the male gender [2]. The most studied function of progesterone in the male system is for the sperm capacitation through a membrane-associated non-genomic signaling pathway [3]. 

Progesterone-induced rapid non-genomic signaling events have been confirmed after molecular cloning of two groups of membrane progesterone receptors [4]. One group is the cytochrome b5-like heme/steroid-binding protein family, including four members, of which only the PGRMC1 protein was confirmed to bind with progesterone [5]. Although PGRMC1 was studied in a variety of human cancers, only one report so far showed its involvement in prostate cancer [6]. The other group is the Class II PAQR family, also known as membrane progestin receptors (mPRs). There are five members in this group: mPRα (PAQR7), mPRβ (PAQR8), mPRγ (PAQR5), mPRδ (PAQR6), and mPRε (PAQR9). Interestingly, only a few studies reported their aberrant expression and genomic abnormality in human cancers, including breast [7], ovarian [8], urinary bladder [9], endometria [10,11], and prostate [12]. 

In the human prostate gland, several progestins, including chlormadinone, and osaterone, were found to reduce the volume of benign prostatic hyperplasia (BPH) mass due to shrinkage of glandular and stromal compartments by antagonizing the AR signaling pathway [13,14]. In prostate cancer tissues, an early study showed PGR expression in less than 50% of tumor cells, which was increased to over 60% in metastatic tissues [13]. However, detailed immunohistochemistry analysis revealed that PGR α-isoform (PR-A) expression was mainly in prostate stromal cells [15], which was reduced, along with higher Gleason scores [16]. Although PGR β-isoform protein (PR-B) exerted a higher expression level in prostate cancer tissues and was significantly correlated with a poor prognosis [17], PGR gene expression at the mRNA levels in prostate cancer tissues was not significantly different from normal prostate tissues [18]. Further analysis with more advanced technology is needed to delineate the exact expression pattern of the PGR gene in prostate cancers. In addition, there is a paucity in the literature for PAQR gene profiles in prostate cancers. 

In this study, we conducted a comprehensive analysis of gene expression profiles for the classic PGR, and the membrane progesterone receptors in prostate cancers at the mRNA levels. We compared their expression levels among different tumor stages or grades and explored their correlation with disease progression and patient survival outcomes. Our results showed that PAQR6 gene expression was significantly upregulated in prostate cancers and correlated with quick disease progression and worse survival outcomes. PAQR6 gene expression was remarkably stimulated by androgen treatment and largely reduced after anti-AR treatment. Expression of PAQR8 and PGRMC2 genes were oppositely associated with NEPC progression and AR signaling in CRPC tissues. These results provided a novel and strong prognostic biomarker for prostate cancer management and new insights of mPR expression profiles in prostate cancers.

## 2. Materials and Methods

### 2.1. Gene Expression Profiles in Benign Prostate Tissues

Cell type-specific gene expression profiles in the prostate gland were assessed with the NCBI GEO dataset GDS1973 [19], which was generated from four different cell types using an antibody pulldown approach against distinct cell surface-specific markers. There were five biological replicates of each pulldown assay, and the expression profiles were determined using the Affymetrix U133 Plus 2.0 arrays. The expression levels of candidate genes were normalized using the β-Actin (ACTB) gene as the internal control.

### 2.2. Gene Expression Profiles in Prostate Cancer LNCaP Cells

Androgen stimulation of gene expression was assessed using the NCBI GEO dataset GDS2782 [20]. Human prostate cancer LNCaP cells (ATCC catalog #CRL-1740) were stimulated with the androgenic hormone dihydrotestosterone (DHT, 1.0 nM) for 16 h in the RPMI1640 media supplied with 10% charcoal-stripped fetal bovine serum. A 2-h pretreatment was conducted with the AR antagonist bicalutamide (CSDX, 10 μM). Labeled mRNAs were hybridized onto oligonucleotide microarrays using the Affymetrix high-density Human Genome U133 Plus 2.0 chips. There were three biological replicates in each treatment group. The expression levels of β-Actin (ACTB) gene were used as the internal control.

### 2.3. Gene Expression Profiles in Primary Prostate Cancers

Gene expression at the mRNA levels in malignant and normal prostate tissues from primary prostate cancer patients was assessed using the Cancer Genome Atlas (TCGA) RNA-seq dataset, which contains molecularly characterized over 20,000 primary cancer and matched normal samples, including prostate cancers [21]. The RNA sequencing data as a value of fragments per kilobase per million (FPKM) were converted to log_2_ value of transcript per million reads (TPM) before subjecting to statistical analysis. In addition to the group comparison of malignant versus normal tissues in 499 cases, a pair-wise comparison was also conducted in 52 case-matched pairs of malignant and normal tissues. Meanwhile, the mRNA expression levels were also assessed using a cDNA microarray dataset generated from 28 normal tissues and 59 malignant tissues derived from a previous report [22]. 

### 2.4. Differences of Gene Expression in Relation to Clinical Parameters

The differences of candidate gene expression were compared in individual pathological stage (TNM category) using the TCGA dataset. Patients were divided into high or low expression groups based on the median expression value of candidate genes. The differences of case incidences were compared between these two groups using Chi-squared tests or Fisher’s exact test. A logistic regression test with one independent variable was conducted to evaluate the odds ratio of the candidate gene between tumor stages or lymph node invasion status. Meanwhile, two more RNA-seq datasets derived from primary prostate cancers were used for assessing the differences of candidate gene expression among different stages, Gleason scores, or relapse situations. One of the datasets was from 112 samples with more than 70% tumor content of primary prostate cancers to ensure tumor transcriptome representativeness [23]. The other dataset was derived from 116 early-onset patients of primary prostate cancers (diagnosis ≤ 55 years) [24]. The correlations between the expression levels of candidate genes and the serum prostate-specific antigen (PSA) levels (the ratio of free-PSA versus total PSA) or relapse interval were assessed using the Spearman coefficient test.

### 2.5. Assessment of Patient Survival Outcomes

Patient survival outcomes, including overall survival, and progression-free interval were assessed using the TCGA dataset with the Kaplan-Meier curve analysis, as described previously [25]. Patients were splatted into high or low expression groups with the minimum *p*-value approach, and the significance of the differences between these groups were statistically analyzed using the Log-rank test. 

### 2.6. Gene Expression Profiles and Correlation Analysis in Metastatic Late-Stage Prostate Cancers

The gene expression profiles in metastatic prostate cancer tissues with castration-resistant phenotype or neuroendocrinal features were assessed using an RNA-seq dataset derived from a previous report [26]. In a total of 195 samples available for gene expression analysis, 89 (45.6%) cases were previously exposed to AR antagonist treatment including Enzalutamide or Abiraterone. The correlations between the expression levels of candidate genes and the AR score, and NEPC score, as well as AR-v7 expression levels, were analyzed using Spearman coefficient analysis. The AR signaling score system was estimated using a gene expression signature from 27 genes that showed robust activation or inhibition of expression upon androgen stimulation [27]. The NEPC score system was established based on 70 genes with drastic differences at the mRNA expression levels or the promotor methylation status between the neuroendocrinal CRPC (CRPC-NE) and castration-resistant adenocarcinoma (CRPC-Adeno) samples, as described previously [28]. 

### 2.7. Data Presentation and Statistical Analysis

Quantitative data were presented as the MEAN with the SEM (standard error of the mean). Differences among multiple groups were analyzed using the ANOVA test, followed by the Kruskal–Wallis. Comparisons between two groups were conducted with the Wilcoxon rank-sum test. Case-matched pair comparison was conducted using the Wilcoxon signed-rank test. The differences in patient survival outcomes between the two subgroups were analyzed using the Kaplan-Meier survival plot using the R survival package (version 3.2-10). Data visualization was conducted using the R survminer package (version 0.4.9). The hazard ratio (HR) was calculated using the Cox regression analysis and tested using the log-rank test.

## 3. Results

### 3.1. Expression Levels of mPR Genes Were Aberrantly Altered in Prostate Cancers

**Figure 1:**
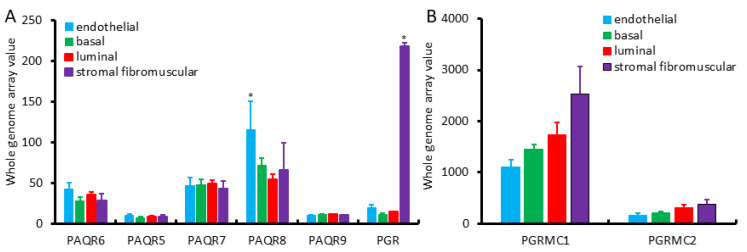
(**A**,**B**) Expression pattern of PAQR genes in different prostate cell types, including basal, luminal secretory, stromal fibromuscular, and endothelial cells of the prostate, as described in a previous report [19]. Cell types were separated with antibody-based magnetic cell sorting (MACS) against distinct cell-type-specific cluster designation (CD) antigens. Gene expression levels were assessed using HG-U133_Plus_2 Affymetrix Human Genome U133 Plus 2.0 Array.

We first assessed the expression profiles of mPR and PGR genes in different cell types of the benign prostate gland using the NCBI GEO dataset GDS1973 [19]. This cDNA microarray dataset was generated on four types of prostatic cells with an antibody-based sorting approach after digesting benign prostate tissues. Antibodies of CD26 (dipeptidyl peptidase IV) were used for luminal cells, CD104 (integrin β4) for basal cells, CD49α (integrin α1) for stromal fibromuscular cells, and CD31 (PECAM-1) for endothelial cells, as described [19]. As shown in Figure 1A, PAQR6/7/8 genes were the major isoforms expressed in all of these cell types with the PAQR8 as the highest expressed gene. In all types of prostatic cells, PAQR5/9 was expressed at a very low level. Both basal and luminal epithelial cells exhibited a similar pattern at a compatible level of all PAQR genes. In contrast, endothelial cells expressed the highest level of the PAQR8 gene. The classic nuclear PGR gene expression was drastically higher in stromal muscular cells with about 10-fold higher than endothelial cells and 20-fold more than epithelial cells, which were in line with previous reports for a predominant stromal expression of the PGR proteins [15,16,17]. PGRMC1 expression was higher in stromal cells than all other three cell types, while PGRMC2 expression was very low than PGRMC1 in all cell types with comparable levels among different cell types (Figure 1B).

**Figure 2:**
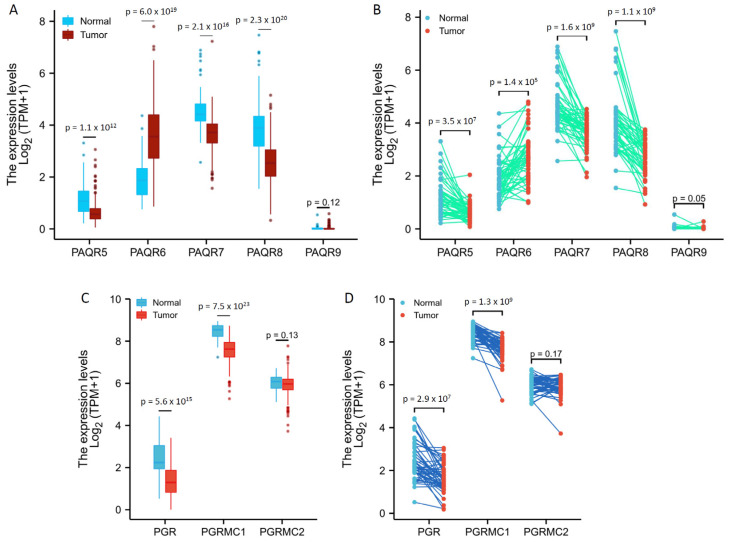
Aberrant expression of PAQR genes in prostate cancers. Gene expression levels were analyzed using the RNA-seq dataset from the TCGA project. Group comparison (**A**,**C**) and pair-wise comparison (**B**,**D**) were conducted in 499 or 52 cases, respectively. The *p*-values were derived from the Wilcoxon rank-sum test for group comparison or Wilcoxon signed-rank test for pair-wise comparison.

We then analyzed the TCGA RNA-seq data for PAQR gene expression profiles in prostate cancers in comparison to benign prostatic tissues. PAQR5/9 were expressed at relatively lower levels in both malignant and benign prostate tissues (Figure 2A,B), confirming the results from the cDNA microarray dataset, as shown in Figure 1. Interestingly, in both group cohort (Figure 2A) and case-matched pair (Figure 2B) comparisons, PAQR6 expression was significantly increased, while PAQR5/7/8 expression was remakably decreased, in malignant tissues compared to benign tissues.

Meanwhile, we analyzed the association of PAQR gene expression with tumor progression in TNM categories. Patients were divided into two groups based on the median level of gene expression. PAQR6 upregulation (PAQR6^high^ cases) was significantly associated with advanced stages (T3–T4) and lymph node invasion (Table 1). Among the downregulated PAQR5/7/8 genes, only PAQR5 (PAQR5^low^ cases), but not PAQR7/8, downregulation was significantly associated with advanced stages (T3–T4) and lymph node invasion (Table 2, Table S1 and Table S2). Single variant logistics analysis revealed that aberrant expression of PAQR5/6 genes was a significant risk factor for tumor stage progression and lymph node invasion (Table 3).

In addition, we analyzed the expression levels of PGR, PGRMC1, and PGRMC2 genes in prostate cancers. As shown in Figure 2C,D, PGR and PGRMC1, but not PGRMC2, expression levels were significantly reduced in malignant tissues compared to normal tissues. However, unlike PAQR5, PGR and PGRMC1 downregulation were not consistently associated with tumor progression in TNM categories, lymph node invasion, and distal metastasis (Table S3 and Table S4). These results suggest a weak clinical significance of PGR or PGRMC1 downregulation in prostate cancers.

**Figure 3:**
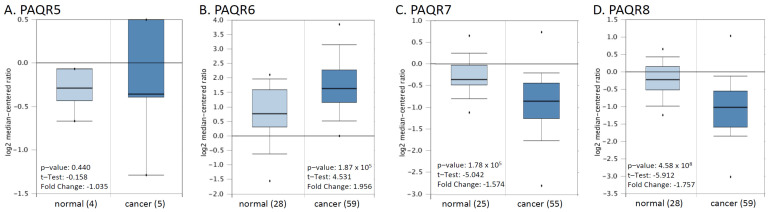
(**A**–**D**) Microarray analysis of PAQR genes in prostate cancers. Expression of PAGR genes was analyzed using the cDNA microarray dataset derived from a previous report [22]. The data figures were generated on the Oncomine platform.

We then verified these alterations using a cDNA microarray dataset from a previous report [22]. As shown in Figure 3, PAQR6 was upregulated about 2-fold, and PAQR7/8 was downregulated more than 1.5-fold in prostate cancers compared to normal prostatic tissues. The alteration for PAQR5 expression was not statistically significant possibly due to less sample size. These results were in line with the RNA-seq data from the TCGA project, indicating a significant alteration of PAQR6/7/8 gene expression in prostate cancer.

### 3.2. PAQR6 Upregulation Is a Strong Prognosis Factor for Disease Progression and Patient Survival

**Figure 4:**
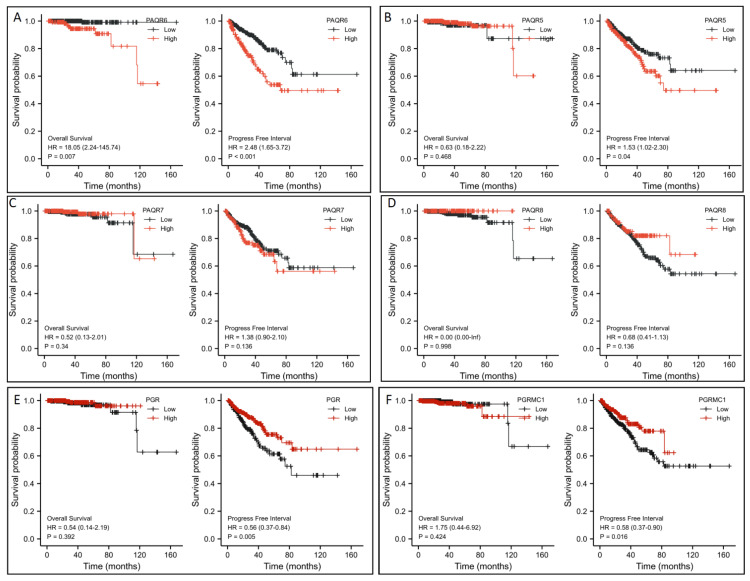
(**A**–**F**) Association of PAQR gene expression with patient survival outcomes in prostate cancers. Patient survival outcomes were analyzed using the TCGA dataset with the Kaplan-Meier plot using the R survival package (version 3.2-10). Data figures were generated using the R survminer package (version 0.4.9). The hazard ratio (HR) was calculated using the Cox regression analysis and tested using the log-rank test.

To assess the clinical significance of this altered gene expression, we first analyzed their impacts on patient survival outcomes. Patients were stratified based on PAQR gene expression level with a minimum *p*-value approach as previously described [25]. Kaplan-Meir survival analysis revealed that PAQR6 upregulation had a significantly negative impact on both overall survival and progression-free interval ((PAQR6^high^ vs. PAQR6^low^; Figure 4A). The hazard ratios were 18.05 for overall survival and 2.48 for the progression-free interval. In contrast, PAQR5/7/8 downregulation had no significant impact on patient survival outcomes (Figure 4B–D). Meanwhile, PGR and PGRMC1 downregulation also had no significant impact on overall survival but a moderate impact on the progression-free interval in prostate cancers (Figure 4E,F). 

**Figure 5:**
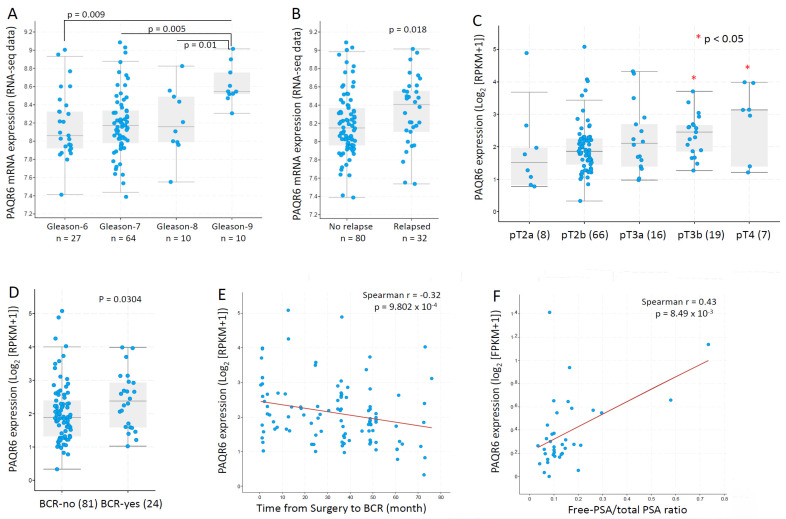
PAQR6 expression was associated with tumor TNM stages, Gleason score, and disease relapse in prostate cancers. PAQ6 expression levels in different patient groups as divided by Gleason scores (panel **A**), disease relapse status (panel **B**), pathological stages (panel **C**), and biochemical relapse (panel **D**) were assessed using the RNA-seq dataset generated from primary prostate cancer tissues [23]. The correlation between PAQR6 expression levels and progression-free interval (panel **E**) or serum PSA levels (panel **F**) were analyzed using the RNA-seq dataset generated from treatment-naïve prostate cancer tissues [29]. The figure images were generated on the cBioportal platform. Case numbers in each group were shown at the bottom of the figures.

We then focused our investigation on PAQR6 and used additional datasets to assess the association of PAQR6 expression with cancer grade and disease progression. In an RNA-seq dataset generated from 112 patients with primary prostate cancers [23], PAQR6 gene expression was gradually elevated along with increasing Gleason scores (Figure 5A). PAQR6 expression was also significantly higher in relapsed patients compared to non-relapsed patients (Figure 5B). In another RNA-seq dataset generated from 116 early-onset prostate cancers (diagnosis ≤ 55 years) [24], a significant increase of PAQR6 gene expression was found in late-stage (pT3b-pT4) patients compared to early-stage (pT2a/b) patients (Figure 5C). PAQR6 expression was also higher in biochemically relapsed patients compared to non-relapse patients (Figure 5D). PAQR6 expression was inversely correlated with a relapse-free interval in this cohort of early-onset patients (Figure 5E). Furthermore, PAQR6 expression was strongly correlated with the clinical biomarker fPSA/tPSA ratio (Figure 5F) in a cohort of 37 treatment-naive prostate cancers [29]. Altogether, these results demonstrated a tight association of PAQR6 upregulation with higher Gleason score, advanced tumor stage, higher fPAS/tPSA ratio, and quick disease relapse in primary prostate cancers. 

**Figure 6:**
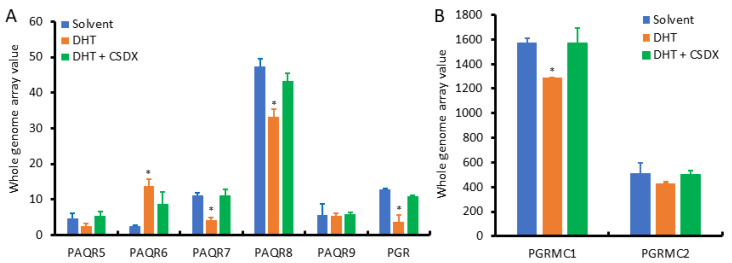
(**A**,**B**) Androgen modulates PAQR gene expression in prostate cancer cells. Prostate cancer LNCaP cells were treated with DHT (1.0 nM) for 16 h with or without Bicalutamide retreatment. Total RNAs were extracted for cDNA microarray with the Affymetrix Human Genome U133 Plus 2.0 Array chips. Data figures were generated using the dataset downloaded from the NCBI GEO dataset GDS2782. The internal normalization was conducted using the expression levels of the ACTB gene. The asterisks indicated a significant difference compared to the solvent control (Student *t*-test, *p* < 0.05).

Since AR signaling is the most critical factor in prostate cancer development and progression, we examined the effect of androgen stimulation on the expression of PAQR genes using the NCBI GEO dataset GDS2782, which was generated in LNCaP cells after dihydrotestosterone (DHT) stimulation plus Bicalutamide blockage for 16 h using the cDNA microarray approach [20]. As shown in Figure 6A, PAQR8 was the predominant isoform at the basal level, followed by PAQR6 and PAQR7. PAQR5 and PAQR9 were the lowest expressed isoforms in LNCaP cells. These expression patterns were in line with the results from prostate tissues (Figure 2A). Interestingly, PAQR6 expression was significantly enhanced by DHT stimulation, which was reversed by AR antagonist Bicalutamide. Conversely, DHT stimulation reduced PAQR7/8, PGR, and PGRMC1 expression (Figure 6B), which was attenuated by Bicalutamide. PAQR5/9 expression was not significantly affected by DHT stimulation. These results indicate that AR signaling positively modulates PAQR6 expression but negatively regulates PAQR7/8 and PGR/PGRMC1 expression in primary prostate cancers.

### 3.3. PAQR6 Expression Is Reduced in Neuroendocrinal Prostate Cancers

**Figure 7:**
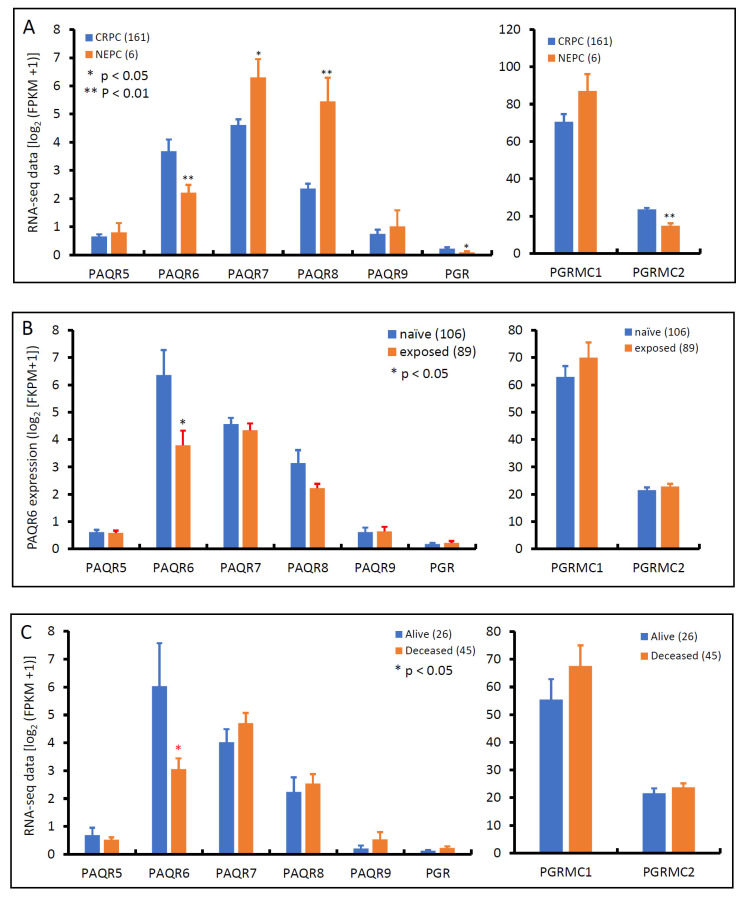
Alterations of PAQR gene expression in CRPC and NEPC tumors. Gene expression levels were compared between CRPC and NEPC tissues (panel **A**), treated with or without AR antagonists (panel **B**), or patients alive or deceased (panel **C**) using the RNA-seq dataset generated from metastatic prostate cancer tissues [26]. Case numbers in each group were shown at the end of figure legends. The *p*-values were derived from the Student *t*-test in comparison to the CRPC, treatment naïve or alive group.

Androgen deprivation therapy is the first-line treatment for metastatic prostate cancers, unfortunately, patients often developed resistance to this therapy, entering a castration-resistant stage, so-called CRPC, without means to cure [30]. After treatment with AR antagonists, such as Enzalutamide and Abiraterone, about 10–17% of CRPC patients evolved into neuroendocrinal transdifferentiated prostate cancer, also known as treatment-induced NEPC [31]. We examined the expression profiles of these progesterone receptor genes in an RNA-seq dataset generated from a cohort of metastatic CRPC/NEPC patients [26]. As shown in Figure 7A, PAQR6 expression was significantly higher in CRPC than NEPC tissues. Conversely, PAQR7/8 was lower in CRPC than NEPC tissues. PAQR5/9 were compatible between CRPC and NEPC tissues. PGR and PGRMC2 gene expression levels were significantly higher in CRPC than in NEPC, but PGRMC1 expression was not statistically different between CRPC and NEPC tumors. AR antagonist treatment significantly lowered PAQR6 expression compared to treatment naïve patients (Figure 7B), consistent with the AR signaling regulation of PAQR6 expression. Meanwhile, PAQR6 levels were significantly lower in tumors derived from deceased patients compared to alive patients (Figure 7C), indicating a correlation of PAQR6 downregulation with NEPC progression and unfavorable survival outcome.

### 3.4. PAQR8 and PGRMC2 Oppositely Correlated with AR Signaling and NE Features

**Figure 8:**
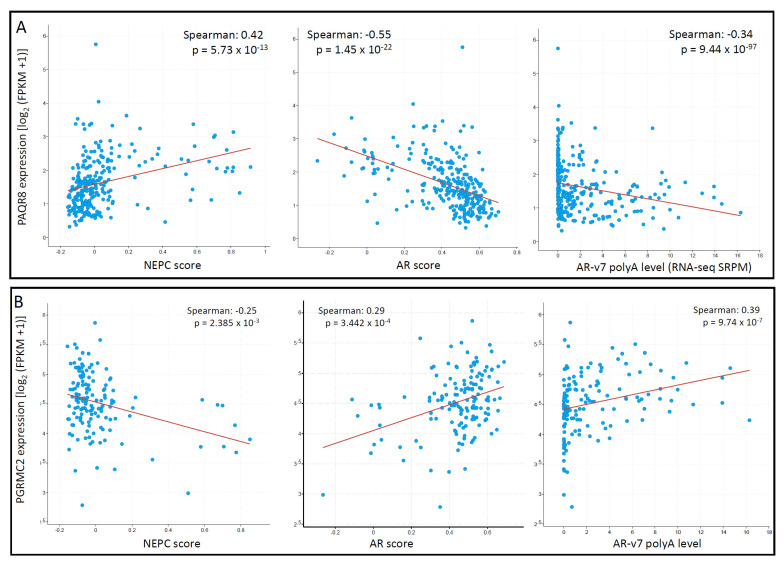
(**A**,**B**) PAQR8 expression is inversely correlated with NEPC and AR scores in prostate cancers. Spearman correlation coefficient analysis was conducted using the RNA-seq dataset generated from metastatic prostate cancers, as described [26]. The figure images were generated on the cBioportal platform.

To explore the correlation of these mPR genes with the critical features, AR and NEPC score, we conducted a Spearman correlation coefficient analysis analyzed in the metastatic CRPC/NEPC cohort [26]. As shown in Figure 8A, PAQR8 expression was strongly correlated with NEPC score but negatively correlated with AR score and AR-v7 expression. In contrast, there was no significant correlations for PAQR5/6/7 genes with NEPC score, AR score, or AR-v7 expression. Although PAQR9 expression negatively correlated with AR score and positively correlated with NEPC score, its expression was very low in more than half of the cases (Supplemental Figure S1), diminishing the importance of these correlations. On the other hand, PGRMC2 expression was negatively correlated with the NEPC score but positively correlated with the AR score and AR-v7 expression levels (Figure 8B). These results suggest that PAQR8 and PGRMC2 were differently modulated by AR signaling during CRPC/NEPC progression. The mechanistic investigation is underway to elucidate their role in prostate cancers.

## 4. Discussion

In this study, we conducted a comprehensive gene expression analysis of seven progesterone receptor genes in primary and late-stage prostate cancer tissues. Our results showed that PAQR6 expression was significantly upregulated in primary prostate cancers. PAQR6 upregulation was associated with cancer grade (Gleason score), tumor stages (TNM categories), disease progression (quick relapse), and survival outcomes. Although PGR, PAQR5/7/8, and PGRMC1 genes were remarkably downregulated in primary prostate cancers, their alteration was not associated with disease progression and patient survival outcomes. These results indicated that PAQR6 is a strong prognosis factor in prostate cancer, possibly modulated by AR signaling.

It is conceivable that AR signaling is the most critical factor in prostate cancer development and progression [30]. In this study, we found that PAQR6 expression was enhanced by DHT stimulation in prostate cancer LNCaP cells, which was blocked by AR antagonist Bicalutamide. PAQR6 expression in patient tumor tissues strongly correlated with clinical biomarker PSA level and associated with biomedical relapse. PAQR6 expression was significantly reduced after AR antagonist treatment, which was further reduced in NEPC patients. These results coordinately demonstrated that PAQR6 expression is positively regulated by AR signaling in prostate cancers. The underlying mechanism is under further investigation by our group.

In comparison between the late-stage CRPC and NEPC tumors, PAQR6, plus PGR/PGRMC2 expression, was reduced, but PAQR7/8 expression was increased in NEPC tumors. We also found a reversal event for PAQR8 downregulation in primary cancers but an upregulation in NEPC tumors. Consistently, PAQR8 expression was negatively associated with AR score and AR-v7 expression but strongly correlated with NEPC score. In LNCaP cells, PAQR8 expression was suppressed by DHT treatment, which was reversed by Bicalutamide. These results indicate that PAQR8 is negatively modulated by AR signaling in prostate cancers, although its clinical significance is under further investigation. However, PGRMC2 expression was in an inverse relationship with PAQR8 and was not stimulated by DHT nor suppressed by AR antagonists, indicating that PGRMC2 might be modulated by a different mechanism during CRPC/NEPC progression from the AR signaling pathway. 

Membrane progesterone receptors (mPRs) are newly discovered genes mediating the non-genomic effect of progesterone in a variety of cell types, including neuronal cells, ovary cells, endometroid cells, and breast cells [32]. Human ovarian cancer cells were the first ones to show mPR activity [8], but the prognostic potential of PAQR7/8 downregulation was established in endometrial cancers in 2018 [11]. High frequency of copy number variation of the PAQR6 gene (60%) was recently found in urinary bladder cancers, which was associated with disease progression in muscle-invasive bladder cancer patients [9]. Similarly, PAQR6 upregulation was reported in prostate cancer tissues and correlated with poor survival outcomes [12]. This report is in line with our results that PAQR6 upregulation is a strong prognostic factor for unfavorable outcomes in prostate cancer patients. In addition, PGRMC1 was reported to distinguish Gleason score 3 versus 4 tumors of prostate cancers, but the clinical significance was not clear [6]. 

Prostate cancers are managed roughly in three steps, early localized diseases are surgically removed by radical prostatectomy or radiation therapy, metastatic diseases are mainly managed by androgen deprivation therapy (ADT), and relapsed diseases after ADT (so-called castration-resistant prostate cancers or CRPC) are treated with a novel generation of anti-AR agents, such as Abiraterone and Enzalutamide [30,33]. In recent years, anti-AR resistant prostate cancer cases with neuroendocrinal features (also known as NEPC) have emerged with an aggressive phenotype in about 17% of CRPC cases [31]. Since progesterone and androgen reside in the same metabolic route, it is reasonable to postulate that inhibiting androgen synthetic enzymes, such as the Abiraterone target CYP17A1, might disturb all related steroid biosynthesis and metabolism, including progesterone [34]. A rapid progression of CRPC patients during palliative treatment with progestins [35] has been reported, indicating a tumor-promoting effect of progestins. Because of the varying expression profiles of individual membrane progesterone receptors in prostate cancers during progression from primary androgen-sensitive to CRPC, eventually to NEPC, it is important to understand the biological responses of individual membrane progesterone receptors before applying progestins as an anti-AR therapy in prostate cancer management.

There were certain limitations in this study, such that the gene expression was assessed only at the mRNA levels but not accompanied with protein levels. We also realized that a mechanistic study is needed to verify the importance of those altered gene expression in prostate cancer progression, especially during the anti-AR treatment-induced neuroendocrinal evolution in CRPC patients. However, this study provided a significant indication for further investigation of PAQR gene regulation during prostate cancer progression.

## 5. Conclusions

Our comprehensive analysis discovered that PAQR6 expression was significantly upregulated in primary prostate cancers, which was strongly correlated with disease progression and patient survival outcomes. As an androgen-regulated gene, PAQR6 is a strong prognostic factor for disease progression and patient survival outcome. On the other hand, PAQR8 expression was negatively regulated by AR signaling and correlated with NEPC progression after ADT/AR antagonist treatment in prostate cancers. Although PGRMC2 expression was positively correlated with AR score and negatively correlated with NEPC score, its expression is potentially modulated by an AR-independent mechanism. 

## Figures and Tables

**Table 1:** Differences of PAQR6 Expression in TNM Categories of Prostate Cancers.

Characteristic	Low PAQR6	High PAQR6	*p*	Statistic	Method
n	249	250			
T stage, n (%)			0.027	7.23	Chisq.test
T2	109 (22.2%)	80 (16.3%)			
T3	132 (26.8%)	160 (32.5%)			
T4	5 (1%)	6 (1.2%)			
N stage, n (%)			0.014	6.08	Chisq.test
N0	179 (42%)	168 (39.4%)			
N1	28 (6.6%)	51 (12%)			
M stage, n (%)			0.249		Fisher.test
M0	224 (48.9%)	231 (50.4%)			
M1	0 (0%)	3 (0.7%)			
Age, median (IQR)	62 (56, 66)	61 (56, 65)	0.327	32701	Wilcoxon

**Table 2:** Differences of PAQR5 Expression in TNM Categories of Prostate Cancers.

Characteristic	Low PAQR5	High PAQR5	*p*	Statistic	Method
n	249	250			
T stage, n (%)			<0.001	19.6	Chisq.test
T2	117 (23.8%)	72 (14.6%)			
T3	128 (26%)	164 (33.3%)			
T4	2 (0.4%)	9 (1.8%)			
N stage, n (%)			0.021	5.31	Chisq.test
N0	171 (40.1%)	176 (41.3%)			
N1	27 (6.3%)	52 (12.2%)			
M stage, n (%)			0.623		Fisher.test
M0	226 (49.3%)	229 (50%)			
M1	2 (0.4%)	1 (0.2%)			
Age, mean ± SD	60.84 ± 7	61.22 ± 6.64	0.529	−0.63	*T* test

**Table 3:** Logistic Analysis of PAQR5/6 in Prostate Cancers.

Characteristics	Total (N)	Odds Ratio (OR)	*p* Value
PAQR5			
T stage (T3 & T4 vs. T2)	492	2.162 (1.495–3.144)	<0.001
N stage (N1 vs. N0)	426	1.871 (1.132–3.152)	0.016
PAQR6			
T stage (T3 & T4 vs. T2)	492	1.651 (1.146–2.387)	0.007
N stage (N1 vs. N0)	426	1.941 (1.177–3.255)	0.01

## Data Availability

The original contributions presented in the study are included in the article/Supplementary Material. Further inquiries can be directed to the corresponding authors.

## Supplementary Materials

The following are available online at https://www.mdpi.com/article/10.3390/biom11091383/s1, Supplemental Figure S1. Correlations of PAQR genes with NEPC score, AR score and AR-v7 expression. Spearman correlations were analyzed using the RNA-seq dataset generated from metastatic prostate cancers, as described [26]. Supplemental Table S1. PAQR7 expression in prostate cancers. Supplemental Table S2. PAQR8 expression in prostate cancers. Supplemental Table S3. PGR expression in prostate cancers. Supplemental Table S4. PGRMC1 expression in prostate cancers.

## Abbreviations

AR	androgen receptor
BCR	Biochemical Relapse
BPH	benign prostate hyperplasia
CRPC	castration-resistant prostate cancer
DHT	dihydrotestosterone
FPKM	fragments per kilobase per million
HR	hazard ratio
mPR	membrane progesterone receptor
NEPC	neuroendocrinal prostate cancer
PGR	progesterone receptor
PGRMC1/2	progesterone receptor membrane component 1/2
PAQR	progestin and adipoQ receptor
PSA	prostate-specific antigen
TCGA	the Cancer Genome Atlas
TPM	transcript per million reads
SEM	standard error of the mean
