# Supplementary material for: PAQR6 Upregulation Is Associated with AR Signaling and Unfavorite Prognosis in Prostate Cancers
Source: Biomolecules. 2021 Sep 18;11(9):1383. doi: 10.3390/biom11091383 (PMC8465620; doi:10.3390/biom11091383)
Supplement: Supplementary file 1 [file biomolecules-11-01383-s001.zip › Figure S1.pdf]

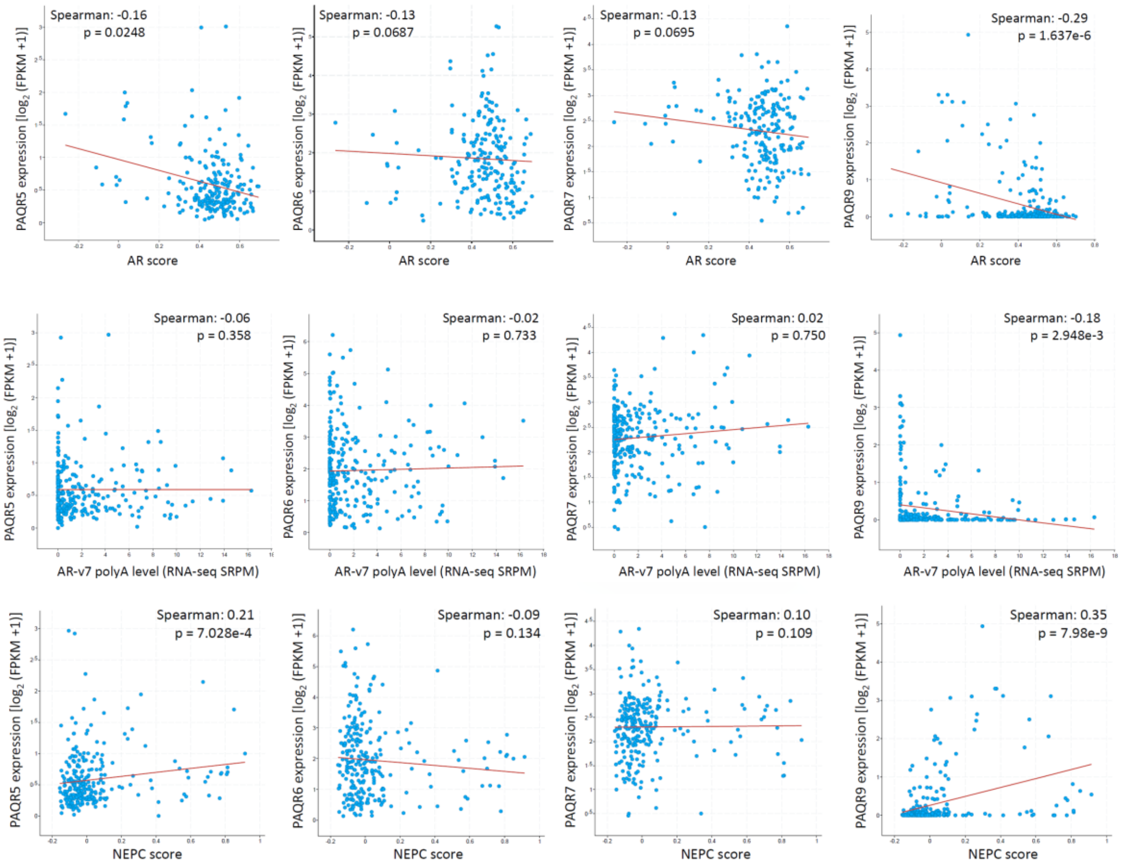

Supplemental Figure S1. Correlations of PAQR genes with NEPC score, AR score and AR-v7 expression. Spearman correlations were analyzed using the RNA-seq dataset generated from metastatic prostate cancers, as described.
