# Supplementary material for: PAQR6 Upregulation Is Associated with AR Signaling and Unfavorite Prognosis in Prostate Cancers
Source: Biomolecules. 2021 Sep 18;11(9):1383. doi: 10.3390/biom11091383 (PMC8465620; doi:10.3390/biom11091383)
Supplement: Supplementary file 1 [file biomolecules-11-01383-s001.zip › Tables S1-S4.pptx]

## Slide 1
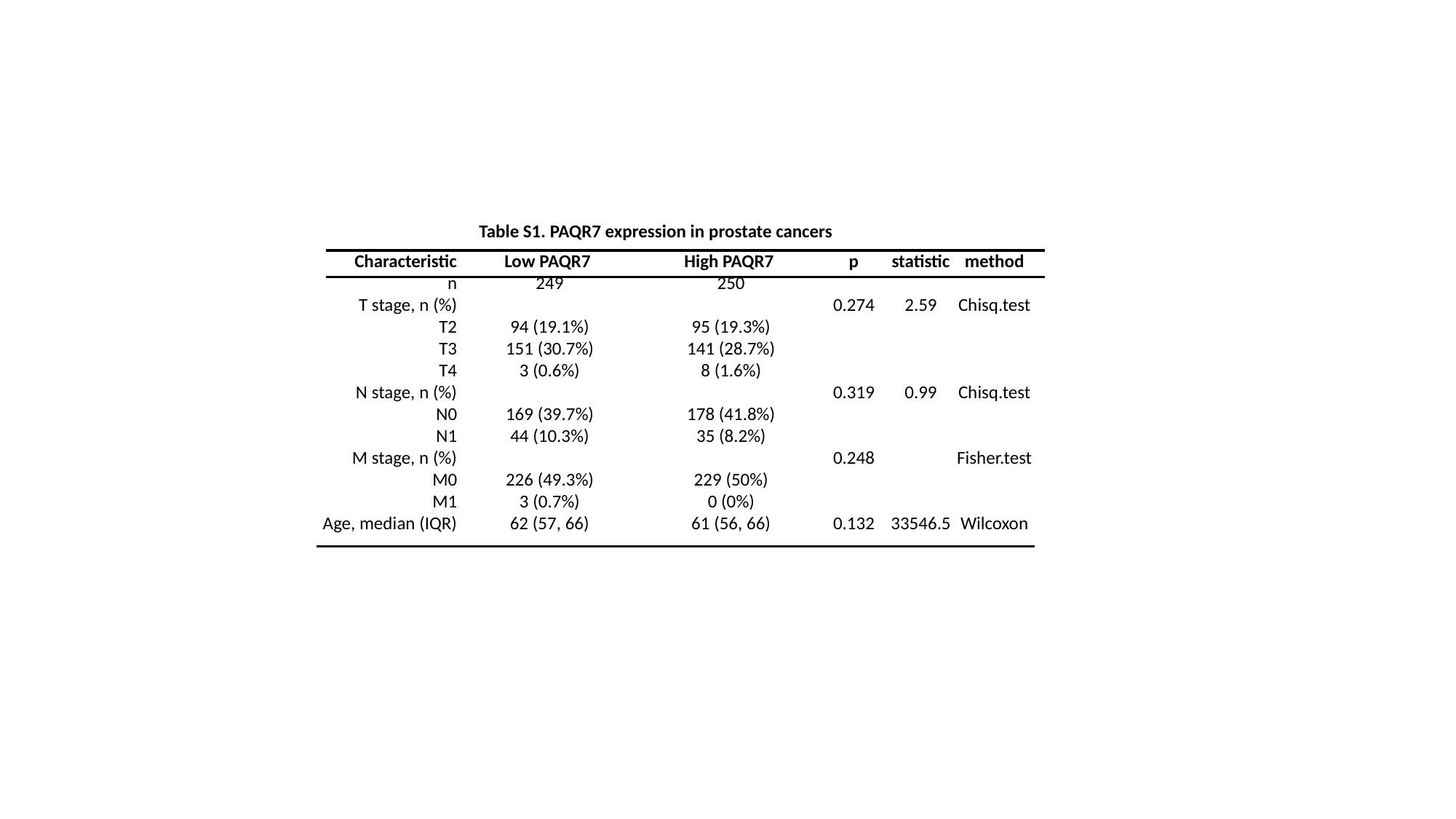

Table S1. PAQR7 expression in prostate cancers
| Characteristic | Low PAQR7 | High PAQR7 | p | statistic | method |
| --- | --- | --- | --- | --- | --- |
| n | 249 | 250 | | | |
| T stage, n (%) | | | 0.274 | 2.59 | Chisq.test |
| T2 | 94 (19.1%) | 95 (19.3%) | | | |
| T3 | 151 (30.7%) | 141 (28.7%) | | | |
| T4 | 3 (0.6%) | 8 (1.6%) | | | |
| N stage, n (%) | | | 0.319 | 0.99 | Chisq.test |
| N0 | 169 (39.7%) | 178 (41.8%) | | | |
| N1 | 44 (10.3%) | 35 (8.2%) | | | |
| M stage, n (%) | | | 0.248 | | Fisher.test |
| M0 | 226 (49.3%) | 229 (50%) | | | |
| M1 | 3 (0.7%) | 0 (0%) | | | |
| Age, median (IQR) | 62 (57, 66) | 61 (56, 66) | 0.132 | 33546.5 | Wilcoxon |

## Slide 2
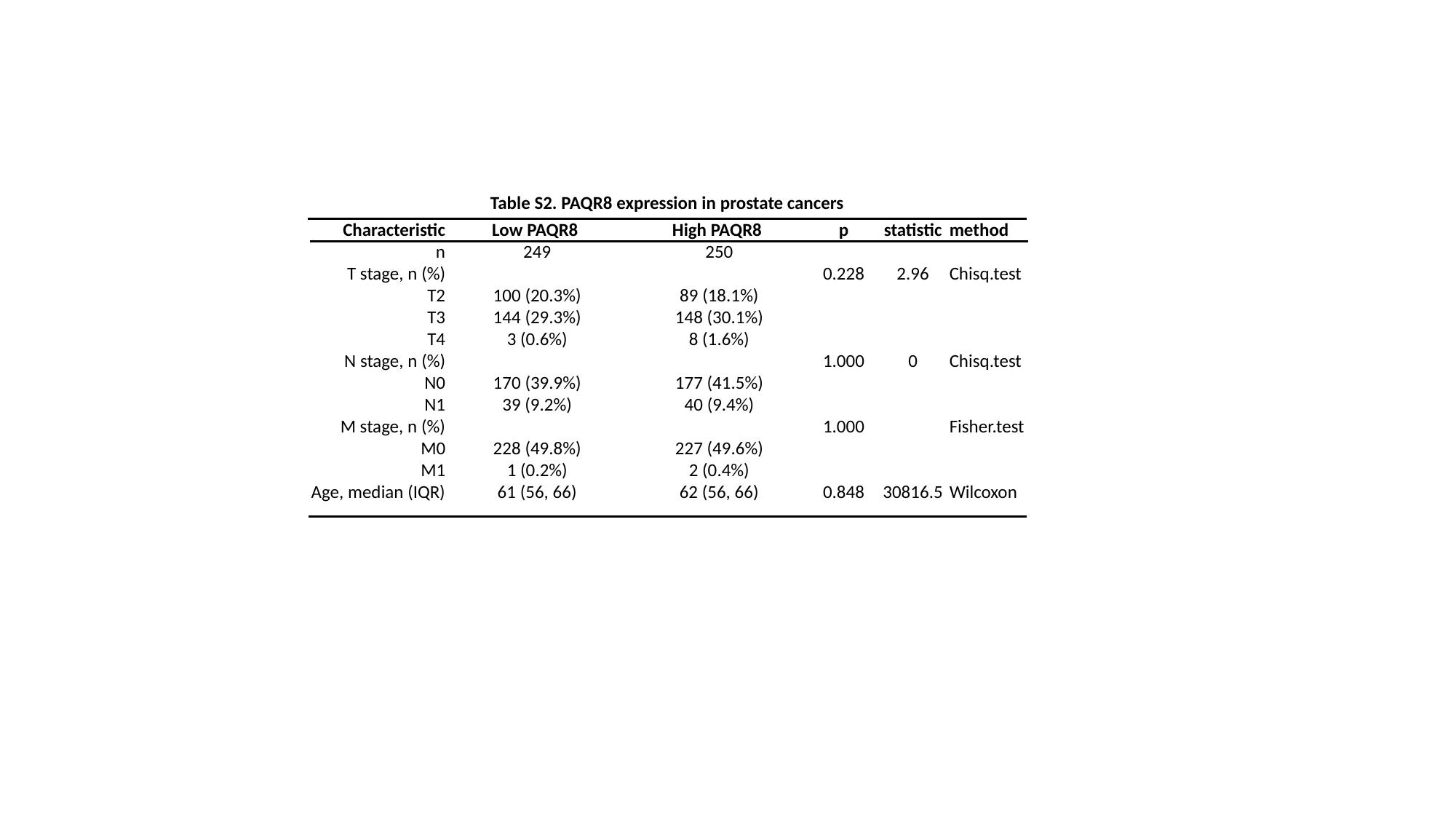

Table S2. PAQR8 expression in prostate cancers
| Characteristic | Low PAQR8 | High PAQR8 | p | statistic | method |
| --- | --- | --- | --- | --- | --- |
| n | 249 | 250 | | | |
| T stage, n (%) | | | 0.228 | 2.96 | Chisq.test |
| T2 | 100 (20.3%) | 89 (18.1%) | | | |
| T3 | 144 (29.3%) | 148 (30.1%) | | | |
| T4 | 3 (0.6%) | 8 (1.6%) | | | |
| N stage, n (%) | | | 1.000 | 0 | Chisq.test |
| N0 | 170 (39.9%) | 177 (41.5%) | | | |
| N1 | 39 (9.2%) | 40 (9.4%) | | | |
| M stage, n (%) | | | 1.000 | | Fisher.test |
| M0 | 228 (49.8%) | 227 (49.6%) | | | |
| M1 | 1 (0.2%) | 2 (0.4%) | | | |
| Age, median (IQR) | 61 (56, 66) | 62 (56, 66) | 0.848 | 30816.5 | Wilcoxon |

## Slide 3
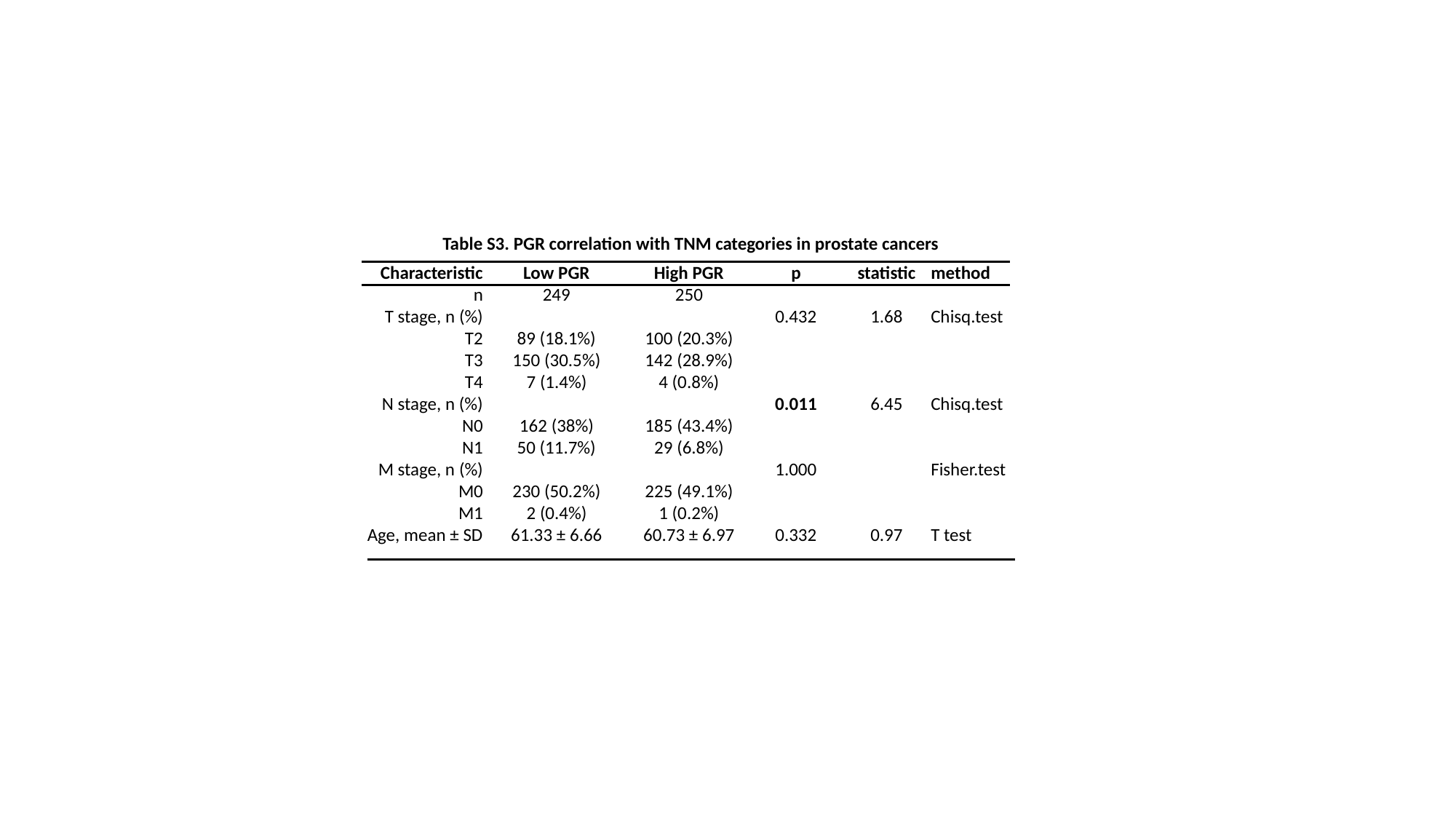

Table S3. PGR correlation with TNM categories in prostate cancers
| Characteristic | Low PGR | High PGR | p | statistic | method |
| --- | --- | --- | --- | --- | --- |
| n | 249 | 250 | | | |
| T stage, n (%) | | | 0.432 | 1.68 | Chisq.test |
| T2 | 89 (18.1%) | 100 (20.3%) | | | |
| T3 | 150 (30.5%) | 142 (28.9%) | | | |
| T4 | 7 (1.4%) | 4 (0.8%) | | | |
| N stage, n (%) | | | 0.011 | 6.45 | Chisq.test |
| N0 | 162 (38%) | 185 (43.4%) | | | |
| N1 | 50 (11.7%) | 29 (6.8%) | | | |
| M stage, n (%) | | | 1.000 | | Fisher.test |
| M0 | 230 (50.2%) | 225 (49.1%) | | | |
| M1 | 2 (0.4%) | 1 (0.2%) | | | |
| Age, mean ± SD | 61.33 ± 6.66 | 60.73 ± 6.97 | 0.332 | 0.97 | T test |

## Slide 4
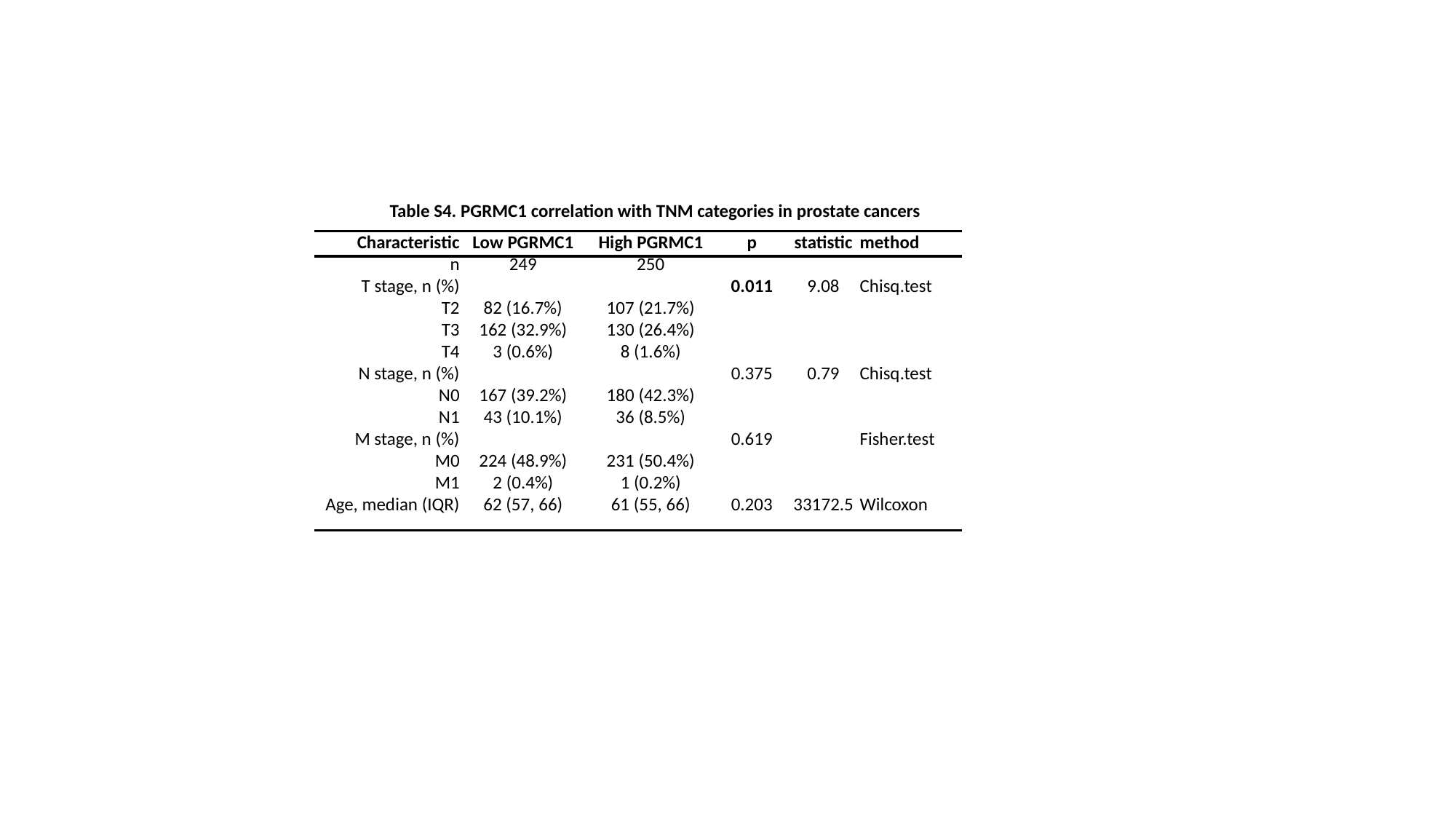

Table S4. PGRMC1 correlation with TNM categories in prostate cancers
| Characteristic | Low PGRMC1 | High PGRMC1 | p | statistic | method |
| --- | --- | --- | --- | --- | --- |
| n | 249 | 250 | | | |
| T stage, n (%) | | | 0.011 | 9.08 | Chisq.test |
| T2 | 82 (16.7%) | 107 (21.7%) | | | |
| T3 | 162 (32.9%) | 130 (26.4%) | | | |
| T4 | 3 (0.6%) | 8 (1.6%) | | | |
| N stage, n (%) | | | 0.375 | 0.79 | Chisq.test |
| N0 | 167 (39.2%) | 180 (42.3%) | | | |
| N1 | 43 (10.1%) | 36 (8.5%) | | | |
| M stage, n (%) | | | 0.619 | | Fisher.test |
| M0 | 224 (48.9%) | 231 (50.4%) | | | |
| M1 | 2 (0.4%) | 1 (0.2%) | | | |
| Age, median (IQR) | 62 (57, 66) | 61 (55, 66) | 0.203 | 33172.5 | Wilcoxon |
